# This is no “ICA bug”: response to the article, “ICA's bug: how ghost ICs emerge from effective rank deficiency caused by EEG electrode interpolation and incorrect re-referencing”

**DOI:** 10.3389/fnimg.2023.1331404

**Published:** 2023-12-21

**Authors:** Arnaud Delorme, Scott Makeig

**Affiliations:** ^1^Swartz Center for Computational Neuroscience, Institute for Neural Computation, University of California, San Diego, San Diego, CA, United States; ^2^CERCO CNRS, UMR 5549, Paul Sabatier University, Toulouse, France

**Keywords:** ICA, EEG, rank, ghost, reference

Data decomposition by Independent Component Analysis (ICA) is commonly applied to biophysical and neurophysiological data to remove artifacts and/or separate brain source activity, for example, in electroencephalographic (EEG) and fMRI data (Makeig et al., 1995; McKeown et al., 1998). ICA takes a data matrix as input (EEG time courses or fMRI maps) to extract component “activation” (component-time course for EEG or component maps for fMRI) defined by an “unmixing” matrix. By taking the inverse of the unmixing matrix, the original data matrix can then be expressed as a linear combination of these component “activations.”

However, ICA, as a blind source separation method, should not be applied blindly. There are several assumptions necessary to justify applying independent component analysis (ICA) to given data.

- First, ICA assumes that the recorded signals are a linear mixture of source signals that are statistically independent (for EEG, temporally independent; for fMRI, spatially dependent). In practice, when applied to EEG or similar data, ICA can extract components representing brain and non-brain (artifact) sources that are maximally but not absolutely independent (Makeig et al., 1995; Delorme et al., 2012). Similarly, when applied to fMRI data, the component voxel maps need not be perfectly independent.
- Second, successful ICA decomposition requires that the source signals (or map weights) are not Gaussian distributed. This is usually not an issue for biological data since physiological and neurophysiological source processes are not expected to have perfectly Gaussian probability density distributions.
- Third, ICA can separate sources whose spatial projection patterns (for EEG) or time courses (for fMRI) remain fixed in the data.
- Finally, most ICA algorithms expect that the number of sources contributing to the data is at least equal to the number of channels provided as input, and that each channel is not a linear mixture of data in other channels.

Let us provide an example for this last assumption. Suppose one has two EEG source signals recorded in three scalp channels (each a linear mixture of the two sources). Here, ICA decomposition will attempt to find the same number of components as the number of channels given as input (i.e., three components), though as the input data only contains two sources, it will prove unable to do so. Similarly, when there are three sources, but one of the three channels is a linear combination of the other two channels (for example, their difference), then ICA decomposition will also fail.

In both cases, we say that the data matrix is rank-deficient: the activity of at least one channel is a linear combination of the other channel. To fix that issue, one of the channels could be dropped, or PCA could be applied to reduce the input data dimensionality, for example, by removing the PCA components with the smallest strengths in the data (i.e., the smallest eigenvalues). However, without such preprocessing, most ICA algorithms (and, in particular, the Infomax ICA algorithm used by Kim et al., 2023) will fail, as they are not designed to handle rank-deficient data.

However, these facts lead Kim et al. (2023) to claim that the Infomax ICA algorithm contains a “bug,” and to claim that the bug appears when decomposing re-referenced or interpolated EEG data. Here, the term “ICA bug” is misleading. When the raw data are first re-referenced or interpolated in preparation for ICA decomposition, as described in Kim et al. (2023), they are made rank deficient. For example, in the common average reference procedure, at each data sample, the sum of all channels is subtracted from each channel. The sum of all channels at any sample is thus 0, for example, with 3 channels A, B, and C, then at each time point A + B + C = 0. This makes the data rank deficient, as each channel is equal to minus the sum of all others (for example, A = –B – C), and Infomax ICA was not designed to process rank-deficient data. Note that this or any *correctly-performed* channel re-referencing may reduce the data rank. This result is not dependent on incorrect re-referencing, as suggested by Kim et al. (2023).

Kim et al. (2023) point out that the function that checks the rank of the data before running Infomax ICA may give inaccurate results, possibly leading to ICA decomposition being applied to rank-deficient matrices This will yield what they term “ghost components”—a fact they refer to as an ICA “bug.” However, the function that fails to estimate the rank of the data correctly is not a part of the ICA algorithm. As we show below, this function may fail to detect that the data are rank deficient (or, effectively rank deficient) because of inherent digital rounding errors and/or MATLAB implementation issues.

Many scientific calculations require using real numbers with high precision, but digital computers can only represent these numbers with a finite number of bits. Single-precision floating-point arithmetic uses 32 bits to represent a number (1 bit for the sign, 8 bits for the exponent, and 23 bits for the mantissa). Double-precision floating-point arithmetic uses 64 bits to represent a number (1 bit for the sign, 11 for the exponent, and 52 for the mantissa). Double-precision arithmetic provides higher precision and a wider range of representable numbers than single-precision arithmetic, but requires more computer memory and other hardware resources. Using single-precision arithmetic can produce rounding errors, truncation errors, and other numerical instabilities that can significantly affect the accuracy and reliability of many scientific computations, including digital filtering (Akbarpour and Tahar, 2007). However, double-precision arithmetic is only less often immune to this problem.

To assess how rank computation is affected by numerical precision, we used publicly available data from an auditory oddball task comprising 39 64-channel data files from 13 subjects, each subject performing three runs [dataset *ds003061* on *nemar.org* (Delorme et al., 2022)]. We imported the raw data, converted it to double precision, removed non-EEG channels and then filtered the EEG data above 0.5 Hz. Here we used the default Hamming windowed, zero-phase, and non-causal *sinc* FIR filter in the *Firfilt* plug-in (v2.6) in EEGLAB (Delorme and Makeig, 2004). Filter order was 1,691 points; transition bandwidth, 0.5 Hz. Next, we converted the data to average reference by (at each time point) subtracting the all-channels mean from each channel, thereby reducing the data rank by 1 and thus making the matrix rank deficient—and thereby unsuitable for ICA decomposition.

We then assessed whether it was possible to detect that the data matrix was no longer full rank (using MATLAB, 2022b running on the Expanse HPC resource). Applying PCA decomposition, the smallest eigenvalue, which should theoretically be 0, was not exactly 0 because of rounding errors introduced by performing the digital arithmetic. Across the 39 data files, the least eigenvalues were 0.057 ± 0.08 for single precision data and 0.0051 ± 0.0098 for double precision data. We then performed a parametric sign test to assess whether these results for single and double-precision computation differed systematically. This showed that the eigenvalues of the double-precision data were systematically (*p* < 10^−11^) closer to 0 than for the single-precision data.

Data rank can also be determined using a second method that applies PCA to the channel covariance matrix. In this case, the least eigenvalues were smaller (0.023 ± 0.11 for single precision, 6^*^10^−9^ ± 2.8^*^10^−9^ for double precision). Again, a sign test showed that the computed eigenvalues of the double-precision data were systematically (*p* < 10^−11^) closer to 0 than for the single-precision data. Thus, applying PCA to the covariance matrix provided more accurate results, especially when applied to double-precision data. As an important note, these numbers proved inconsistent across platforms and MATLAB versions. While the mean for single precision data of the first method was 0.057 on the Expanse HPC resource (MATLAB, 2022b), it was 0.01 on a Fedora core Xeon workstation (MATLAB, 2023a), and 0.11 on Macbook Pro M1 (MATLAB, 2023b). Smaller differences were also observed across the double-precision results. This highlights the well-established fact that numerical precision varies across platforms and numerical libraries.

Kim et al. (2023) argue that the function detecting rank-deficient matrices that raises an issue for EEG data decomposition is the *pop_runica* function of the EEGLAB software package—which calculates the rank of the (double precision) data and if needed reduces its dimensionality using PCA (i.e., when the input data are not full rank), then calls the Infomax ICA decomposition function *runica*. The *pop_runica* function calculates rank by applying the two methods described above to the first 3,000 samples of the data (to reduce computation time).

In the first method using the MATLAB *rank* function, data rank is computed by performing a singular value decomposition of the data and then counting the number of eigenvalues that exceed a threshold (the data- and computer-dependent numerical precision of the maximum eigenvalue times the number of EEG data samples). This strategy often fails when applied to EEG data recordings. For example, it fails on the 39 EEG recordings above, returning a rank of 64 instead of 63 when the data are double precision. Using all the data points (instead of the first 3,000 as in the *pop_runica* function) returns the same result. Surprisingly, applied to the same data in single-precision, the same rank function returns the correct data rank (63) for 23 of the 39 datasets, but dramatically underestimates the rank for the other 16. The second MATLAB rank computation method, computed from the eigenvalues of the covariance matrix, uses a fixed threshold of 10^−7^. This method accurately estimated the rank (63) for all 39 EEG recordings above in double and single precision.

In its original implementation, the EEGLAB *pop_runica* function selected the maximum of the ranks computed using the two methods described above. Kim et al. (2023) proposed the use of the *minimum* of the two values, since (as for the example data treated here) the first method tends to *overestimate* data rank. As of September 2023 (EEGLAB 2021.1), the EEGLAB *pop_runica* function does use this minimum to estimate data rank.

EEGLAB users should note that the EEGLAB tutorials do not rely on the accuracy of the Matlab *rank* function, as problems associated with computing the rank of EEG data have been known for more than a decade, and users are advised to input explicitly the rank of the input EEG matrix, as can typically be inferred from the operations performed on the raw data (e.g., data re-referencing or scalp channel interpolation) in preparation for ICA decomposition.

In conclusion, although Kim et al. (2023) did propose a useful data rank estimation improvement (in *pop_runica*), the title of their report is misleading to potential ICA users—this is *not* an “ICA bug.”

## Author contributions

AD: Writing—original draft, Writing—review & editing. SM: Writing—review & editing.

## References

[B1] Akbarpour B. Tahar S. (2007). Error analysis of digital filters using HOL theorem proving. J. Appl. Logic 5, 651–666. 10.1016/j.jal.2006.11.001

[B2] Delorme A. Makeig S. (2004). EEGLAB: an open source toolbox for analysis of single-trial EEG dynamics including independent component analysis. J. Neurosci. Methods 134, 9–21. 10.1016/j.jneumeth.2003.10.009 15102499

[B3] Delorme A. Palmer J. Onton J. Oostenveld R. Makeig S. (2012). Independent EEG sources are dipolar. PLoS ONE 7, e30135. 10.1371/journal.pone.0030135 22355308 PMC3280242

[B4] Delorme A. Truong D. Youn C. Sivagnanam S. Stirm C. Yoshimoto K.. (2022). NEMAR: an open access data, tools and compute resource operating on neuroelectromagnetic data. Database 2022:baac096. 10.1093/database/baac096 36367313 PMC9650770

[B5] Kim H. Luo J. Chu S. Cannard C. Hoffmann S. Miyakoshi M. (2023). ICA's bug: How ghost ICs emerge from effective rank deficiency caused by EEG electrode interpolation and incorrect re-referencing. Front. Signal Process. 3, 1064138. 10.3389/frsip.2023.1064138 PMC10764542 38179199

[B6] Makeig S. Bell A. Jung T. P. Sejnowski T. J. (1995). Independent component analysis of electroencephalographic data. Adv. Neural Inf. Process. Sys. 8.

[B7] McKeown M. J. Makeig S. Brown G. G. Jung T. P. Kindermann S. S. Bell A. J.. (1998). Analysis of fMRI data by blind separation into independent spatial components. Hum. Brain Mapp. 6, 160–188. 10.1002/(SICI)1097-0193(1998)6:3<160::AID-HBM5>3.0.CO;2-1 9673671 PMC6873377

